# Supplementary material for: Modeling glioblastoma heterogeneity as a dynamic network of cell states
Source: Mol Syst Biol. 2021 Sep 16;17(9):e10105. doi: 10.15252/msb.202010105 (PMC8444284; doi:10.15252/msb.202010105)
Supplement: Supplementary file 5 — Source Data for Figure 3 [file MSB-17-e10105-s001.zip › Figure3A_sourcedata/GSEA_3065/nosigdb_state1.GseaPreranked.1622718409535/FELDMAN_2020_S-G2.html]

Details for gene set FELDMAN\_2020\_S-G2[GSEA]

|  || Dataset | state1 |
| Phenotype | NoPhenotypeAvailable |
| Upregulated in class | na\_pos |
| GeneSet | FELDMAN\_2020\_S-G2 |
| Enrichment Score (ES) | 0.43950748 |
| Normalized Enrichment Score (NES) | 1.8227782 |
| Nominal p-value | 0.0 |
| FDR q-value | 0.0015631962 |
| FWER p-Value | 0.035 |
Table: GSEA Results Summary

  

Fig 1: Enrichment plot: FELDMAN\_2020\_S-G2      
 Profile of the Running ES Score & Positions of GeneSet Members on the Rank Ordered List

  

| PROBE | GENE SYMBOL | GENE\_TITLE | RANK IN GENE LIST | RANK METRIC SCORE | RUNNING ES | CORE ENRICHMENT || 1 | TMEM158 |  |  | 3 | 0.835 | 0.0145 | Yes |
| 2 | HMGA1 |  |  | 8 | 0.736 | 0.0272 | Yes |
| 3 | IGFBP3 |  |  | 15 | 0.664 | 0.0384 | Yes |
| 4 | UBE2S |  |  | 19 | 0.634 | 0.0493 | Yes |
| 5 | SMS |  |  | 23 | 0.619 | 0.0600 | Yes |
| 6 | MAP3K7CL |  |  | 32 | 0.546 | 0.0689 | Yes |
| 7 | UPP1 |  |  | 37 | 0.523 | 0.0778 | Yes |
| 8 | PTTG1 |  |  | 40 | 0.520 | 0.0868 | Yes |
| 9 | NETO2 |  |  | 41 | 0.517 | 0.0960 | Yes |
| 10 | HOXB2 |  |  | 57 | 0.455 | 0.1025 | Yes |
| 11 | PLP2 |  |  | 59 | 0.446 | 0.1103 | Yes |
| 12 | DCBLD2 |  |  | 62 | 0.444 | 0.1180 | Yes |
| 13 | C12orf75 |  |  | 66 | 0.432 | 0.1254 | Yes |
| 14 | TUBB4B |  |  | 67 | 0.429 | 0.1330 | Yes |
| 15 | PRELID1 |  |  | 77 | 0.406 | 0.1393 | Yes |
| 16 | PHLDA1 |  |  | 83 | 0.389 | 0.1457 | Yes |
| 17 | IGFBP5 |  |  | 88 | 0.385 | 0.1521 | Yes |
| 18 | PMAIP1 |  |  | 93 | 0.380 | 0.1585 | Yes |
| 19 | TNFRSF12A |  |  | 104 | 0.364 | 0.1639 | Yes |
| 20 | PDCD5 |  |  | 109 | 0.358 | 0.1698 | Yes |
| 21 | TUBA1B |  |  | 119 | 0.347 | 0.1751 | Yes |
| 22 | CTNNAL1 |  |  | 123 | 0.343 | 0.1808 | Yes |
| 23 | UBE2C |  |  | 125 | 0.340 | 0.1868 | Yes |
| 24 | FTL |  |  | 134 | 0.333 | 0.1919 | Yes |
| 25 | FHL2 |  |  | 141 | 0.328 | 0.1971 | Yes |
| 26 | H2AFZ |  |  | 153 | 0.321 | 0.2016 | Yes |
| 27 | RANBP1 |  |  | 154 | 0.320 | 0.2073 | Yes |
| 28 | LSM4 |  |  | 162 | 0.316 | 0.2122 | Yes |
| 29 | HMGN2 |  |  | 164 | 0.313 | 0.2177 | Yes |
| 30 | IL11 |  |  | 166 | 0.312 | 0.2231 | Yes |
| 31 | SNRPB |  |  | 172 | 0.310 | 0.2281 | Yes |
| 32 | RAN |  |  | 194 | 0.297 | 0.2312 | Yes |
| 33 | CKS2 |  |  | 195 | 0.297 | 0.2365 | Yes |
| 34 | BIRC5 |  |  | 202 | 0.295 | 0.2411 | Yes |
| 35 | CYCS |  |  | 203 | 0.294 | 0.2463 | Yes |
| 36 | DTYMK |  |  | 205 | 0.292 | 0.2514 | Yes |
| 37 | EIF4EBP1 |  |  | 216 | 0.290 | 0.2555 | Yes |
| 38 | MRPL17 |  |  | 226 | 0.285 | 0.2596 | Yes |
| 39 | ENC1 |  |  | 229 | 0.284 | 0.2645 | Yes |
| 40 | POP7 |  |  | 236 | 0.282 | 0.2688 | Yes |
| 41 | SIVA1 |  |  | 252 | 0.276 | 0.2722 | Yes |
| 42 | SLC25A5 |  |  | 254 | 0.276 | 0.2770 | Yes |
| 43 | CDKN1A |  |  | 262 | 0.274 | 0.2811 | Yes |
| 44 | PIN1 |  |  | 264 | 0.274 | 0.2859 | Yes |
| 45 | RAD23A |  |  | 277 | 0.269 | 0.2894 | Yes |
| 46 | CHCHD2 |  |  | 278 | 0.269 | 0.2942 | Yes |
| 47 | SNRPG |  |  | 287 | 0.264 | 0.2980 | Yes |
| 48 | HPRT1 |  |  | 289 | 0.263 | 0.3026 | Yes |
| 49 | DEK |  |  | 296 | 0.261 | 0.3066 | Yes |
| 50 | MLLT11 |  |  | 321 | 0.254 | 0.3086 | Yes |
| 51 | CKS1B |  |  | 346 | 0.242 | 0.3104 | Yes |
| 52 | PTGES3 |  |  | 359 | 0.237 | 0.3134 | Yes |
| 53 | YWHAH |  |  | 365 | 0.237 | 0.3170 | Yes |
| 54 | PTMA |  |  | 366 | 0.236 | 0.3213 | Yes |
| 55 | SNRNP25 |  |  | 367 | 0.236 | 0.3255 | Yes |
| 56 | PFDN2 |  |  | 370 | 0.234 | 0.3294 | Yes |
| 57 | HSPD1 |  |  | 397 | 0.227 | 0.3307 | Yes |
| 58 | TUBB |  |  | 398 | 0.226 | 0.3347 | Yes |
| 59 | CHCHD3 |  |  | 402 | 0.226 | 0.3384 | Yes |
| 60 | RAB13 |  |  | 417 | 0.223 | 0.3409 | Yes |
| 61 | HNRNPAB |  |  | 430 | 0.221 | 0.3436 | Yes |
| 62 | TUBG1 |  |  | 439 | 0.219 | 0.3467 | Yes |
| 63 | MZT1 |  |  | 449 | 0.216 | 0.3496 | Yes |
| 64 | CACYBP |  |  | 458 | 0.214 | 0.3525 | Yes |
| 65 | MRPL18 |  |  | 470 | 0.212 | 0.3551 | Yes |
| 66 | TMEM106C |  |  | 478 | 0.211 | 0.3581 | Yes |
| 67 | RHEB |  |  | 491 | 0.207 | 0.3606 | Yes |
| 68 | LYPD1 |  |  | 508 | 0.205 | 0.3625 | Yes |
| 69 | H2AFV |  |  | 538 | 0.198 | 0.3630 | Yes |
| 70 | HSPE1 |  |  | 545 | 0.197 | 0.3659 | Yes |
| 71 | TPX2 |  |  | 556 | 0.194 | 0.3683 | Yes |
| 72 | DCTPP1 |  |  | 566 | 0.191 | 0.3707 | Yes |
| 73 | CALM2 |  |  | 597 | 0.186 | 0.3709 | Yes |
| 74 | AKAP12 |  |  | 605 | 0.185 | 0.3735 | Yes |
| 75 | ANP32B |  |  | 622 | 0.183 | 0.3750 | Yes |
| 76 | EIF1AX |  |  | 624 | 0.182 | 0.3782 | Yes |
| 77 | SET |  |  | 634 | 0.181 | 0.3805 | Yes |
| 78 | SNRPD1 |  |  | 647 | 0.179 | 0.3824 | Yes |
| 79 | PSMC3 |  |  | 692 | 0.173 | 0.3809 | Yes |
| 80 | SNRPE |  |  | 707 | 0.171 | 0.3824 | Yes |
| 81 | CMC2 |  |  | 722 | 0.169 | 0.3840 | Yes |
| 82 | TMEM237 |  |  | 735 | 0.167 | 0.3857 | Yes |
| 83 | HMGN1 |  |  | 737 | 0.167 | 0.3885 | Yes |
| 84 | STMN1 |  |  | 763 | 0.163 | 0.3888 | Yes |
| 85 | CENPN |  |  | 764 | 0.163 | 0.3917 | Yes |
| 86 | PA2G4 |  |  | 780 | 0.160 | 0.3930 | Yes |
| 87 | EIF5 |  |  | 793 | 0.158 | 0.3945 | Yes |
| 88 | UBE2I |  |  | 834 | 0.153 | 0.3931 | Yes |
| 89 | PGP |  |  | 852 | 0.151 | 0.3940 | Yes |
| 90 | CDCA2 |  |  | 859 | 0.150 | 0.3960 | Yes |
| 91 | SAE1 |  |  | 863 | 0.150 | 0.3983 | Yes |
| 92 | CMTM7 |  |  | 868 | 0.148 | 0.4006 | Yes |
| 93 | PSMG1 |  |  | 871 | 0.148 | 0.4030 | Yes |
| 94 | C6orf15 |  |  | 921 | 0.143 | 0.4004 | Yes |
| 95 | TUBB6 |  |  | 931 | 0.142 | 0.4020 | Yes |
| 96 | ANLN |  |  | 941 | 0.141 | 0.4035 | Yes |
| 97 | POLR3K |  |  | 952 | 0.140 | 0.4050 | Yes |
| 98 | FOSL1 |  |  | 953 | 0.139 | 0.4075 | Yes |
| 99 | ZFAND6 |  |  | 957 | 0.139 | 0.4096 | Yes |
| 100 | WDR34 |  |  | 972 | 0.137 | 0.4106 | Yes |
| 101 | GGCT |  |  | 974 | 0.137 | 0.4129 | Yes |
| 102 | SAC3D1 |  |  | 976 | 0.137 | 0.4152 | Yes |
| 103 | CEP55 |  |  | 986 | 0.135 | 0.4167 | Yes |
| 104 | CDCA3 |  |  | 994 | 0.134 | 0.4183 | Yes |
| 105 | HMGB1 |  |  | 995 | 0.134 | 0.4207 | Yes |
| 106 | RPL34 |  |  | 1023 | 0.131 | 0.4202 | Yes |
| 107 | SNRPA1 |  |  | 1032 | 0.130 | 0.4217 | Yes |
| 108 | RPA3 |  |  | 1039 | 0.129 | 0.4234 | Yes |
| 109 | MAGOHB |  |  | 1047 | 0.128 | 0.4249 | Yes |
| 110 | RPL22L1 |  |  | 1048 | 0.128 | 0.4272 | Yes |
| 111 | SSRP1 |  |  | 1051 | 0.128 | 0.4293 | Yes |
| 112 | TK1 |  |  | 1056 | 0.127 | 0.4311 | Yes |
| 113 | COPS3 |  |  | 1059 | 0.127 | 0.4332 | Yes |
| 114 | SRSF3 |  |  | 1076 | 0.125 | 0.4337 | Yes |
| 115 | HNRNPDL |  |  | 1086 | 0.124 | 0.4350 | Yes |
| 116 | RFC2 |  |  | 1094 | 0.123 | 0.4364 | Yes |
| 117 | NUDT1 |  |  | 1144 | 0.118 | 0.4334 | Yes |
| 118 | CDKN2D |  |  | 1213 | 0.111 | 0.4282 | Yes |
| 119 | CMSS1 |  |  | 1229 | 0.110 | 0.4286 | Yes |
| 120 | EIF2S1 |  |  | 1243 | 0.109 | 0.4292 | Yes |
| 121 | CENPU |  |  | 1249 | 0.108 | 0.4306 | Yes |
| 122 | BRIX1 |  |  | 1254 | 0.108 | 0.4321 | Yes |
| 123 | CENPW |  |  | 1278 | 0.106 | 0.4316 | Yes |
| 124 | KIF4A |  |  | 1303 | 0.103 | 0.4309 | Yes |
| 125 | PRDX1 |  |  | 1305 | 0.103 | 0.4326 | Yes |
| 126 | TFDP1 |  |  | 1318 | 0.102 | 0.4332 | Yes |
| 127 | HSPB11 |  |  | 1325 | 0.101 | 0.4343 | Yes |
| 128 | EEF2 |  |  | 1328 | 0.101 | 0.4359 | Yes |
| 129 | CKAP2L |  |  | 1359 | 0.099 | 0.4345 | Yes |
| 130 | CENPV |  |  | 1361 | 0.098 | 0.4362 | Yes |
| 131 | MAD2L1 |  |  | 1394 | 0.095 | 0.4345 | Yes |
| 132 | DHFR |  |  | 1413 | 0.094 | 0.4343 | Yes |
| 133 | CTGF |  |  | 1416 | 0.094 | 0.4358 | Yes |
| 134 | UBE2T |  |  | 1440 | 0.093 | 0.4350 | Yes |
| 135 | NXT1 |  |  | 1448 | 0.093 | 0.4359 | Yes |
| 136 | ZWILCH |  |  | 1449 | 0.092 | 0.4375 | Yes |
| 137 | DEPDC1 |  |  | 1478 | 0.090 | 0.4362 | Yes |
| 138 | H2AFX |  |  | 1479 | 0.090 | 0.4378 | Yes |
| 139 | TYMS |  |  | 1501 | 0.089 | 0.4372 | Yes |
| 140 | HNRNPD |  |  | 1509 | 0.088 | 0.4380 | Yes |
| 141 | NCL |  |  | 1512 | 0.088 | 0.4394 | Yes |
| 142 | CENPE |  |  | 1545 | 0.086 | 0.4376 | Yes |
| 143 | TEX30 |  |  | 1547 | 0.086 | 0.4390 | Yes |
| 144 | HMGB2 |  |  | 1570 | 0.085 | 0.4382 | Yes |
| 145 | PXMP2 |  |  | 1573 | 0.085 | 0.4395 | Yes |
| 146 | DNAJC9 |  |  | 1594 | 0.083 | 0.4389 | No |
| 147 | HIST1H1E |  |  | 1608 | 0.082 | 0.4390 | No |
| 148 | FBL |  |  | 1628 | 0.080 | 0.4384 | No |
| 149 | KIF23 |  |  | 1638 | 0.080 | 0.4389 | No |
| 150 | PBK |  |  | 1651 | 0.079 | 0.4390 | No |
| 151 | WDR83OS |  |  | 1667 | 0.077 | 0.4388 | No |
| 152 | COMMD6 |  |  | 1699 | 0.076 | 0.4369 | No |
| 153 | TOMM40 |  |  | 1705 | 0.075 | 0.4377 | No |
| 154 | SPDL1 |  |  | 1731 | 0.073 | 0.4364 | No |
| 155 | RBM17 |  |  | 1733 | 0.073 | 0.4376 | No |
| 156 | NUSAP1 |  |  | 1736 | 0.073 | 0.4387 | No |
| 157 | SNRNP40 |  |  | 1750 | 0.072 | 0.4386 | No |
| 158 | CENPA |  |  | 1788 | 0.070 | 0.4360 | No |
| 159 | PRC1 |  |  | 1794 | 0.070 | 0.4367 | No |
| 160 | PLAU |  |  | 1801 | 0.069 | 0.4373 | No |
| 161 | CCNA2 |  |  | 1835 | 0.068 | 0.4351 | No |
| 162 | CKLF |  |  | 1837 | 0.068 | 0.4362 | No |
| 163 | EXOSC8 |  |  | 1851 | 0.067 | 0.4360 | No |
| 164 | KIF22 |  |  | 1858 | 0.067 | 0.4366 | No |
| 165 | TCF19 |  |  | 1866 | 0.067 | 0.4370 | No |
| 166 | ST13 |  |  | 1885 | 0.066 | 0.4363 | No |
| 167 | IER2 |  |  | 1899 | 0.065 | 0.4361 | No |
| 168 | USP1 |  |  | 1921 | 0.064 | 0.4351 | No |
| 169 | ZWINT |  |  | 1923 | 0.064 | 0.4361 | No |
| 170 | ATP6V1G1 |  |  | 1946 | 0.063 | 0.4349 | No |
| 171 | TACC3 |  |  | 1949 | 0.063 | 0.4358 | No |
| 172 | CDCA4 |  |  | 2073 | 0.056 | 0.4239 | No |
| 173 | MIS18A |  |  | 2094 | 0.055 | 0.4228 | No |
| 174 | GMNN |  |  | 2097 | 0.055 | 0.4236 | No |
| 175 | BEX1 |  |  | 2118 | 0.054 | 0.4224 | No |
| 176 | KPNB1 |  |  | 2179 | 0.052 | 0.4171 | No |
| 177 | RBBP8 |  |  | 2212 | 0.051 | 0.4146 | No |
| 178 | RGCC |  |  | 2214 | 0.051 | 0.4154 | No |
| 179 | CENPH |  |  | 2215 | 0.051 | 0.4163 | No |
| 180 | BTG1 |  |  | 2251 | 0.049 | 0.4135 | No |
| 181 | SMC4 |  |  | 2308 | 0.047 | 0.4085 | No |
| 182 | TRIP13 |  |  | 2314 | 0.047 | 0.4088 | No |
| 183 | ACYP1 |  |  | 2324 | 0.046 | 0.4087 | No |
| 184 | RNPS1 |  |  | 2347 | 0.045 | 0.4072 | No |
| 185 | CENPM |  |  | 2348 | 0.045 | 0.4080 | No |
| 186 | FEN1 |  |  | 2420 | 0.043 | 0.4013 | No |
| 187 | PAICS |  |  | 2456 | 0.041 | 0.3984 | No |
| 188 | RPL39L |  |  | 2461 | 0.041 | 0.3987 | No |
| 189 | BTG3 |  |  | 2514 | 0.039 | 0.3939 | No |
| 190 | LRR1 |  |  | 2521 | 0.039 | 0.3940 | No |
| 191 | HNRNPF |  |  | 2541 | 0.038 | 0.3927 | No |
| 192 | MYBL2 |  |  | 2549 | 0.038 | 0.3926 | No |
| 193 | HSP90AA1 |  |  | 2562 | 0.038 | 0.3920 | No |
| 194 | GINS2 |  |  | 2567 | 0.038 | 0.3923 | No |
| 195 | DNMT1 |  |  | 2576 | 0.037 | 0.3921 | No |
| 196 | GTSE1 |  |  | 2703 | 0.033 | 0.3795 | No |
| 197 | HIST1H1C |  |  | 2712 | 0.033 | 0.3792 | No |
| 198 | RPS27L |  |  | 2723 | 0.032 | 0.3788 | No |
| 199 | DBF4 |  |  | 2751 | 0.031 | 0.3765 | No |
| 200 | MKI67 |  |  | 2760 | 0.031 | 0.3762 | No |
| 201 | CKAP2 |  |  | 2763 | 0.031 | 0.3765 | No |
| 202 | PSMC3IP |  |  | 2766 | 0.031 | 0.3769 | No |
| 203 | ARHGAP11A |  |  | 2814 | 0.030 | 0.3725 | No |
| 204 | CARHSP1 |  |  | 2827 | 0.030 | 0.3718 | No |
| 205 | PFDN5 |  |  | 2857 | 0.029 | 0.3693 | No |
| 206 | FBXO5 |  |  | 2870 | 0.028 | 0.3685 | No |
| 207 | ASF1B |  |  | 2929 | 0.027 | 0.3629 | No |
| 208 | RRS1 |  |  | 2969 | 0.026 | 0.3593 | No |
| 209 | NUDT15 |  |  | 2985 | 0.025 | 0.3581 | No |
| 210 | DUT |  |  | 3005 | 0.025 | 0.3566 | No |
| 211 | DERA |  |  | 3045 | 0.024 | 0.3529 | No |
| 212 | TMPO |  |  | 3093 | 0.023 | 0.3484 | No |
| 213 | DCK |  |  | 3176 | 0.021 | 0.3402 | No |
| 214 | HAUS1 |  |  | 3219 | 0.020 | 0.3362 | No |
| 215 | VRK1 |  |  | 3288 | 0.018 | 0.3294 | No |
| 216 | SKA3 |  |  | 3384 | 0.016 | 0.3197 | No |
| 217 | CDK1 |  |  | 3396 | 0.016 | 0.3188 | No |
| 218 | PCNA |  |  | 3540 | 0.013 | 0.3041 | No |
| 219 | HIST1H1D |  |  | 3604 | 0.012 | 0.2977 | No |
| 220 | SHCBP1 |  |  | 3637 | 0.011 | 0.2945 | No |
| 221 | RFC5 |  |  | 3697 | 0.010 | 0.2885 | No |
| 222 | SRSF2 |  |  | 3783 | 0.008 | 0.2798 | No |
| 223 | HJURP |  |  | 3796 | 0.008 | 0.2787 | No |
| 224 | YPEL5 |  |  | 3803 | 0.008 | 0.2782 | No |
| 225 | DONSON |  |  | 4072 | 0.003 | 0.2502 | No |
| 226 | KLF6 |  |  | 4117 | 0.002 | 0.2456 | No |
| 227 | C4orf3 |  |  | 4151 | 0.002 | 0.2422 | No |
| 228 | HIRIP3 |  |  | 4163 | 0.001 | 0.2410 | No |
| 229 | NCAPG |  |  | 4261 | -0.000 | 0.2309 | No |
| 230 | SUPT16H |  |  | 4275 | -0.001 | 0.2295 | No |
| 231 | EPS8L1 |  |  | 4289 | -0.001 | 0.2282 | No |
| 232 | HSPA8 |  |  | 4292 | -0.001 | 0.2280 | No |
| 233 | NUF2 |  |  | 4342 | -0.002 | 0.2229 | No |
| 234 | SRSF7 |  |  | 4413 | -0.003 | 0.2156 | No |
| 235 | RNASEH2B |  |  | 4493 | -0.004 | 0.2074 | No |
| 236 | MELK |  |  | 4558 | -0.006 | 0.2008 | No |
| 237 | MCM3 |  |  | 4633 | -0.007 | 0.1932 | No |
| 238 | CENPK |  |  | 4697 | -0.007 | 0.1867 | No |
| 239 | SPC25 |  |  | 4740 | -0.008 | 0.1824 | No |
| 240 | AURKB |  |  | 4764 | -0.008 | 0.1802 | No |
| 241 | BUB3 |  |  | 4828 | -0.010 | 0.1738 | No |
| 242 | TMEM178A |  |  | 4968 | -0.012 | 0.1594 | No |
| 243 | MND1 |  |  | 4982 | -0.012 | 0.1583 | No |
| 244 | KIFC1 |  |  | 5017 | -0.013 | 0.1549 | No |
| 245 | MXD3 |  |  | 5096 | -0.014 | 0.1470 | No |
| 246 | HIST1H1B |  |  | 5125 | -0.014 | 0.1443 | No |
| 247 | KIF20B |  |  | 5169 | -0.015 | 0.1401 | No |
| 248 | DSN1 |  |  | 5258 | -0.016 | 0.1312 | No |
| 249 | CCDC34 |  |  | 5298 | -0.017 | 0.1274 | No |
| 250 | TRA2B |  |  | 5321 | -0.017 | 0.1254 | No |
| 251 | CISD2 |  |  | 5358 | -0.018 | 0.1219 | No |
| 252 | KPNA2 |  |  | 5461 | -0.020 | 0.1116 | No |
| 253 | RMI2 |  |  | 5517 | -0.020 | 0.1062 | No |
| 254 | PSIP1 |  |  | 5794 | -0.025 | 0.0777 | No |
| 255 | CLSPN |  |  | 5848 | -0.026 | 0.0726 | No |
| 256 | RTKN2 |  |  | 5868 | -0.027 | 0.0711 | No |
| 257 | PARP2 |  |  | 5986 | -0.029 | 0.0594 | No |
| 258 | DIAPH3 |  |  | 6103 | -0.031 | 0.0478 | No |
| 259 | RRM2 |  |  | 6214 | -0.033 | 0.0368 | No |
| 260 | ARL6IP5 |  |  | 6216 | -0.033 | 0.0373 | No |
| 261 | NDC80 |  |  | 6240 | -0.034 | 0.0355 | No |
| 262 | EEF1A1 |  |  | 6584 | -0.040 | 0.0003 | No |
| 263 | BEX4 |  |  | 6702 | -0.043 | -0.0112 | No |
| 264 | DDIT4 |  |  | 6713 | -0.043 | -0.0115 | No |
| 265 | C19orf48 |  |  | 6811 | -0.045 | -0.0208 | No |
| 266 | RNASEH2A |  |  | 6891 | -0.047 | -0.0283 | No |
| 267 | CENPF |  |  | 6902 | -0.047 | -0.0285 | No |
| 268 | HIST2H2AC |  |  | 7036 | -0.051 | -0.0415 | No |
| 269 | SMC3 |  |  | 7149 | -0.054 | -0.0523 | No |
| 270 | LMNB1 |  |  | 7166 | -0.055 | -0.0530 | No |
| 271 | MCM7 |  |  | 7397 | -0.061 | -0.0760 | No |
| 272 | RRM1 |  |  | 7556 | -0.065 | -0.0914 | No |
| 273 | CEP78 |  |  | 7627 | -0.067 | -0.0975 | No |
| 274 | TOP2A |  |  | 7678 | -0.069 | -0.1015 | No |
| 275 | ARL6IP6 |  |  | 7747 | -0.072 | -0.1074 | No |
| 276 | CDC45 |  |  | 7751 | -0.072 | -0.1064 | No |
| 277 | ASPM |  |  | 7821 | -0.074 | -0.1123 | No |
| 278 | PARP1 |  |  | 7864 | -0.076 | -0.1154 | No |
| 279 | TUBA1A |  |  | 7955 | -0.080 | -0.1234 | No |
| 280 | HAT1 |  |  | 7958 | -0.080 | -0.1222 | No |
| 281 | BRCA2 |  |  | 8192 | -0.089 | -0.1450 | No |
| 282 | EIF4A2 |  |  | 8210 | -0.090 | -0.1452 | No |
| 283 | RAD51AP1 |  |  | 8227 | -0.091 | -0.1453 | No |
| 284 | MIS18BP1 |  |  | 8274 | -0.093 | -0.1484 | No |
| 285 | RFC4 |  |  | 8292 | -0.094 | -0.1485 | No |
| 286 | ESCO2 |  |  | 8566 | -0.109 | -0.1752 | No |
| 287 | SESN3 |  |  | 8632 | -0.113 | -0.1800 | No |
| 288 | MAGED2 |  |  | 8642 | -0.114 | -0.1789 | No |
| 289 | GGH |  |  | 8643 | -0.114 | -0.1769 | No |
| 290 | SMC1A |  |  | 8681 | -0.116 | -0.1787 | No |
| 291 | SMC2 |  |  | 8721 | -0.119 | -0.1807 | No |
| 292 | SDCBP |  |  | 8790 | -0.124 | -0.1856 | No |
| 293 | HIST1H4C |  |  | 8820 | -0.126 | -0.1864 | No |
| 294 | ORC6 |  |  | 8888 | -0.133 | -0.1910 | No |
| 295 | ATAD2 |  |  | 8948 | -0.138 | -0.1948 | No |
| 296 | ATAD5 |  |  | 9000 | -0.142 | -0.1976 | No |
| 297 | PLD3 |  |  | 9093 | -0.151 | -0.2045 | No |
| 298 | CDH2 |  |  | 9187 | -0.163 | -0.2113 | No |
| 299 | RNF19A |  |  | 9358 | -0.191 | -0.2258 | No |
| 300 | SYNE2 |  |  | 9362 | -0.193 | -0.2226 | No |
| 301 | CNPY2 |  |  | 9373 | -0.195 | -0.2202 | No |
| 302 | HELLS |  |  | 9406 | -0.201 | -0.2200 | No |
| 303 | RABAC1 |  |  | 9430 | -0.207 | -0.2187 | No |
| 304 | NASP |  |  | 9437 | -0.208 | -0.2157 | No |
| 305 | TM7SF2 |  |  | 9450 | -0.213 | -0.2131 | No |
| 306 | ITM2B |  |  | 9486 | -0.223 | -0.2128 | No |
| 307 | BAMBI |  |  | 9499 | -0.225 | -0.2101 | No |
| 308 | HES4 |  |  | 9536 | -0.234 | -0.2097 | No |
| 309 | PRKDC |  |  | 9539 | -0.236 | -0.2057 | No |
| 310 | LAPTM4A |  |  | 9553 | -0.241 | -0.2028 | No |
| 311 | TMEM59 |  |  | 9573 | -0.249 | -0.2003 | No |
| 312 | IFITM3 |  |  | 9643 | -0.277 | -0.2026 | No |
| 313 | NDFIP1 |  |  | 9647 | -0.280 | -0.1980 | No |
| 314 | PCSK1N |  |  | 9695 | -0.309 | -0.1974 | No |
| 315 | PLP1 |  |  | 9702 | -0.314 | -0.1924 | No |
| 316 | SOX4 |  |  | 9704 | -0.318 | -0.1869 | No |
| 317 | MIAT |  |  | 9732 | -0.341 | -0.1836 | No |
| 318 | SPARC |  |  | 9738 | -0.347 | -0.1780 | No |
| 319 | PSAP |  |  | 9742 | -0.351 | -0.1721 | No |
| 320 | SOX9 |  |  | 9754 | -0.358 | -0.1669 | No |
| 321 | ID3 |  |  | 9778 | -0.389 | -0.1623 | No |
| 322 | CLU |  |  | 9795 | -0.418 | -0.1566 | No |
| 323 | SAT1 |  |  | 9807 | -0.445 | -0.1498 | No |
| 324 | GPM6A |  |  | 9820 | -0.499 | -0.1422 | No |
| 325 | S100B |  |  | 9822 | -0.507 | -0.1333 | No |
| 326 | FABP7 |  |  | 9825 | -0.513 | -0.1244 | No |
| 327 | PLAT |  |  | 9833 | -0.550 | -0.1153 | No |
| 328 | SCD5 |  |  | 9838 | -0.576 | -0.1055 | No |
| 329 | PRCP |  |  | 9855 | -0.712 | -0.0945 | No |
| 330 | MALAT1 |  |  | 9868 | -0.912 | -0.0796 | No |
| 331 | PTPRZ1 |  |  | 9869 | -0.918 | -0.0632 | No |
| 332 | TTYH1 |  |  | 9872 | -1.033 | -0.0451 | No |
| 333 | GPM6B |  |  | 9873 | -1.091 | -0.0257 | No |
| 334 | SCRG1 |  |  | 9879 | -1.473 | -0.0000 | No |
Table: GSEA details [plain text format]

  

Fig 2: FELDMAN\_2020\_S-G2: Random ES distribution      
 Gene set null distribution of ES for **FELDMAN\_2020\_S-G2**
